# The Multidrug Resistance Protein OsMDR4 Is Involved in Cadmium Absorption in Rice (*Oryza sativa* L.)

**DOI:** 10.3390/plants15152378

**Published:** 2026-08-03

**Authors:** Zijing Xie, Xiaohua Hao, Dan Zhao, Han Lei, Xinzhou Jin, Sha Wu, Wenli Hu, Lianfu Tian, Dongping Li

**Affiliations:** 1 Hunan Provincial Key Laboratory of the Traditional Chinese Medicine Agricultural Biogenomics, Changsha Medical University, Changsha 410219, China; 2 Hunan Province Key Laboratory of Crop Sterile Germplasm Resource Innovation and Application, College of Life Sciences, Hunan Normal University, Changsha 410081, China; 3 College of Life and Environmental Science, Hunan University of Arts and Science, Changde 415000, China

**Keywords:** rice, ABC transporter, OsMDR4, cadmium accumulation

## Abstract

Cadmium (Cd) is a toxic metal that poses a significant threat to crop production and global food security. Transporters play a critical role in mediating the uptake of metal ions, including Cd. However, a substantial number of Cd transporters in rice remain uncharacterized. In this study, we identify OsMDR4, a member of the multidrug resistance protein family, as a mediator of Cd uptake in rice. Heterologous overexpression of OsMDR4 in yeast increased both Cd sensitivity and intracellular Cd accumulation. Consistent with this, the Cd concentrations in both roots and shoots of the *mdr4* mutants were significantly lower than those in wild type. Kinetic analysis further revealed that the maximum Cd uptake rate in *mdr4* mutants was markedly reduced compared with wild type. Expression analysis showed that *OsMDR4* is primarily expressed in the epidermis and root hairs of rice seedlings and in floral organs during the flowering stage. Notably, *OsMDR4* expression in seedling roots was upregulated in response to Cd exposure. Subcellular localization analysis revealed that OsMDR4–EGFP was predominantly localized to the plasma membrane in a heterologous Arabidopsis protoplast system. In summary, we have identified and characterized OsMDR4 as a previously uncharacterized protein that contributes to cadmium accumulation in rice.

## 1. Introduction

Cd is a non-essential heavy metal that poses a serious threat to both human health and crop safety. Excess Cd accumulation in the body is associated with a range of severe diseases, including prostate and lung cancers, testicular damage, and renal tubular injury [1,2].

In plants, excess Cd similarly inhibits growth and development [3]. Cd stress significantly reduces root length, shoot length, and biomass accumulation in rice seedlings, accompanied by decreased chlorophyll content and disrupted mineral element metabolism [4]. In soybean (*Glycine max* L.), Cd exposure causes dose-dependent reductions in root length and shoot length, along with substantial declines in photosynthetic pigment content and antioxidant capacity [5]. Similar inhibitory effects have been reported in cucumber, where Cd stress suppresses growth parameters and induces oxidative damage, resulting in reduced leaf area and biomass [6]. Collectively, these findings indicate that Cd toxicity can severely constrain plant growth, although species vary considerably in their Cd uptake, detoxification, sequestration, and tolerance mechanisms.

Rice (*Oryza sativa* L.), a monocotyledonous staple food crop, exhibits a particularly high propensity for Cd accumulation [7]. Over the past decades, substantial efforts have been devoted to understanding the mechanisms underlying Cd absorption and translocation in rice [8]. Consequently, a series of genes involved in Cd transport have been identified and characterized.

In rice, the uptake of Cd from soil solution into roots is mediated by several transport systems that normally function in the acquisition of essential micronutrients, such as iron (Fe), manganese (Mn), and zinc (Zn). Among these, OsNRAMP5 serves as the primary pathway for Cd entry into rice roots, and OsNRAMP5 overexpression reduces Cd accumulation in grain by promoting Cd sequestration in root vacuoles or by altering root-to-shoot partitioning, whereas loss of function reduces Cd uptake at the root surface. Both manipulations can result in lower grain Cd but through distinct mechanisms [9,10,11]. OsNRAMP1 and OsNRAMP5 exhibit distinct tissue expression patterns and substrate preferences; although they share some functional overlap, OsNRAMP1 does not fully compensate for the loss of OsNRAMP5, particularly in root Cd uptake. In addition, OsIRT1 and OsIRT2 contribute to Cd uptake under iron-deficient conditions [12], and several other transporters have been implicated in Cd translocation and distribution [13,14,15]. While these studies have established a framework for understanding Cd transport in rice, the molecular components involved in this process are not yet fully elucidated. In particular, the roles of many transporter families, including the ATP-binding cassette (ABC) transporter superfamily, remain largely unexplored in the context of Cd uptake.

The ABC transporter superfamily is one of the largest and most evolutionarily conserved transporter families, facilitating the transport of diverse substrates across biological membranes [16]. In plants, ABC transporters are classified into several subfamilies, among which the multidrug resistance (MDR) proteins constitute the largest full-molecule subfamily [17]. In Arabidopsis, ABC transporters have been shown to participate in various physiological processes, including auxin transport, heavy metal homeostasis, and stress responses [18,19,20,21]. In rice, 17 putative MDR genes have been identified [22]; however, most rice MDR members remain functionally uncharacterized. Among the few functionally studied members, OsMDR13/OsABCB14 has been reported to regulate cellular auxin and iron homeostasis [23]; OsABCB4 modulates heading date, pollen fertility, and grain filling through auxin homeostasis, as its disruption delays heading and compromises multiple agronomic traits [24]; and OsMDR7/OsABCB5 enhances cold tolerance via auxin signaling [25]. Despite these advances, whether rice MDR transporters are involved in heavy metal transport, particularly Cd uptake and accumulation, has not yet been reported.

To identify novel transporters involved in Cd uptake in rice roots, we identified Cd-induced transporter genes expressed in rice roots through transcriptome analysis. Among eight candidate genes selected for further analysis, we focused on *OsMDR4* (Os01g0290700) [22], a root-expressed multidrug resistance transporter with unknown function. In this study, we generated CRISPR/Cas9-knockout mutants of *OsMDR4* and characterized their role in Cd accumulation through a combination of yeast heterologous expression, Cd uptake kinetics, promoter-GUS analysis, and subcellular localization. Our findings reveal that OsMDR4 is a previously uncharacterized plasma membrane-localized protein that contributes to Cd accumulation in rice roots, providing new insights into the molecular network governing Cd transport in rice.

## 2. Results

### 2.1. OsMDR4 Contributes to Cd Accumulation

**Figure 1:**
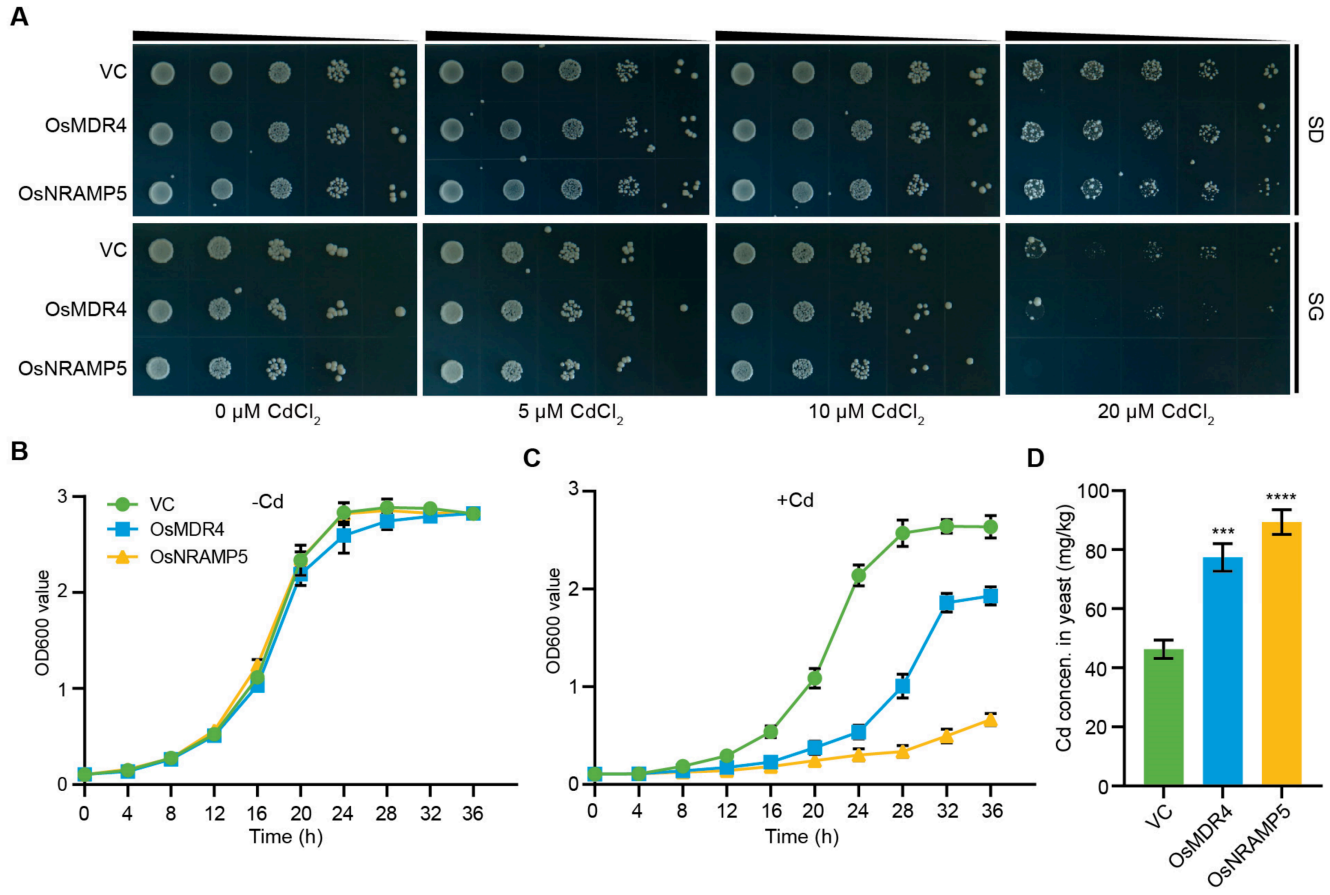
Cadmium sensitivity analysis of OsMDR4 expressed in yeast. (**A**) Growth of yeast strain *Δycf1* expressing OsMDR4, OsNRAMP5, or empty vector control in medium containing different concentrations of cadmium. (**B**) Growth curves plotted from OD600 values in liquid cultures without cadmium. (**C**) Growth curves plotted from OD600 values in liquid medium with 20 μM cadmium. (**D**) Cadmium concentrations of yeast strain *Δycf1* expressing OsMDR4, OsNRAMP5 or vector control after 24 h of culture in liquid medium with 10 μM cadmium. Each point represents the mean ± SD from three independent biological replicates, each derived from independent yeast transformations grown as separate cultures. Values represent means ± SD (n = 3) from three biological replicates. Statistical significance of the difference between the empty vector control and *OsMDR4-* or *OsNRAMP5*-expressing strains was determined by Student’s *t*-test (*** *p* < 0.001; **** *p* < 0.0001).

To investigate OsMDR4 function in a heterologous yeast system, we expressed OsMDR4 in the *Δycf1* yeast mutant. OsNRAMP5, a known Cd import transporter [9,10,11], served as a positive control. Initially, *Δycf1* yeast strains harboring *pYES2*, *OsMDR4-pYES2* or *OsNRAMP5-pYES2* were spotted onto SD or SG medium plates containing varying concentrations of Cd. Yeast colonies expressing OsMDR4, OsNRAMP5, or empty vector showed comparable growth on SD medium with different Cd concentrations (Figure 1A). However, when expression was induced on SG medium with 20 μM Cd, the growth of yeast expressing OsMDR4 or OsNRAMP5 was impaired (Figure 1A). This observation was further supported by growth curve analysis, which demonstrated that *Δycf1* yeast expressing OsMDR4 or OsNRAMP5 grew slower than those expressing empty vector under 20 μM Cd treatment (Figure 1B,C). We hypothesize that excessive Cd accumulation in cells leads to impaired yeast growth. Cd concentration measurements revealed that the Cd concentration in yeast expressing OsMDR4 or OsNRAMP5 was 1.67-fold and 1.93-fold higher, respectively, compared with the vector control (Figure 1D).

Additionally, a Cd-specific fluorescent probe was used to visualize the Cd level in yeast cells. The green fluorescence intensity in yeast cells transformed with OsMDR4 or OsNRAMP5 was significantly stronger than that in cells transformed with the empty vector (Figure S1).

These results suggest that OsMDR4 contributes to Cd accumulation in cells.

### 2.2. Long-Term Cd Accumulation in Wild-Type and mdr4 Mutants

**Figure 2:**
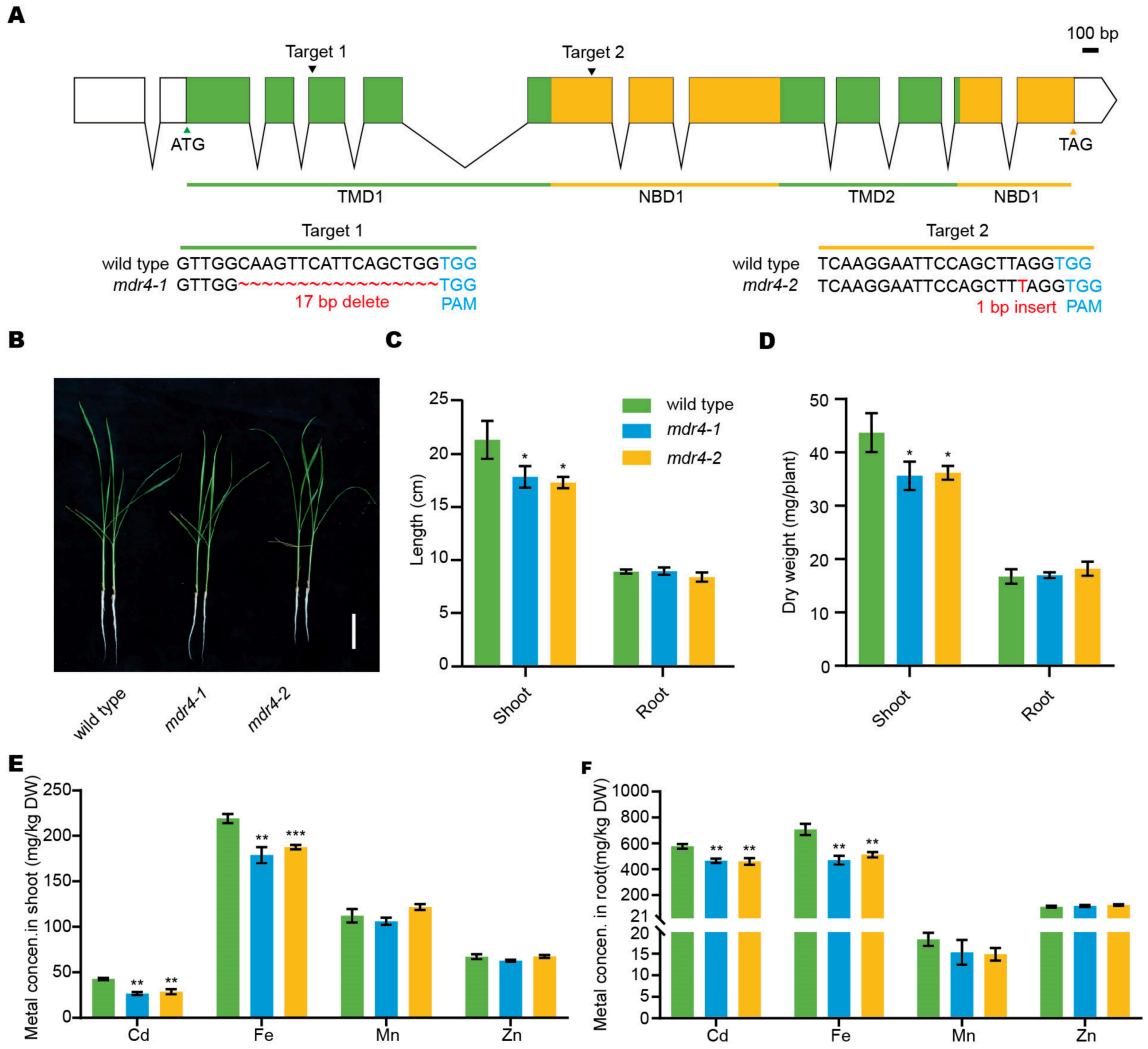
CRISPR/Cas9-based mutation of OsMDR4 and phenotypic analysis of *mdr4* mutants seedling. (**A**) Schematic representation of the gene model of *OsMDR4* and DNA sequences of the CRISPR/Cas9-edited *OsMDR4* gene; the arrow indicates the gRNA target site; the 20 bp guide RNA (gRNA) spacer sequence for the Cas9/gRNA complex is shown in black, and the protospacer adjacent motif (PAM) site is shown in blue; deleted nucleotides and inserted nucleotides are shown in red. The lengths of the insertions and/or deletions (In/Del) are shown. (**B**) The growth phenotype of wild type and *mdr4* mutants under half strength of Kimura’s B for 3 weeks, scale bar = 5 cm. (**C**) Root and shoot length of wild type and *mdr4* mutants; plants grown in half strength of Kimura’s B for 3 weeks. (**D**) Root and shoot dry weight of wild type and *mdr4* mutants; plants grown in half strength of Kimura’s B for 3 weeks. (**E**,**F**) Metal concentrations in shoot or root of wild type and *mdr4* mutants; plants grown in half strength of Kimura’s B for for 3 weeks and then transferred into half strength of Kimura’s B with 5 μM cadmium for a week. For each experiment, the two CRISPR alleles (*mdr4-1* and *mdr4-2*) were analyzed independently. Data from the two mutants are presented separately in all figures. Statistical comparisons were performed separately for each mutant relative to wild-type controls. Values represent means ± SD (n = 3) from three biological replicates. Statistical significance of the difference between the wild type and *mdr4* mutants was determined by a *t*-test (* *p* < 0.05; ** *p* < 0.01; *** *p* < 0.001).

As a typical full-molecule transporter, OsMDR4 consists of two transmembrane domains (TMDs) and two nucleotide binding domains (NBDs) (Figure S2) [26]. To determine the function of OsMDR4 in rice, two targets located in the first TMD and NBD were selected to generate two independent *mdr4* mutants in the Nipponbare background using the CRISPR/Cas9 system (Figure 2A). Sequencing analysis showed a 17 bp nucleotide deletion in the first target, designated as *mdr4-1*, and a 1 bp insertion in the second target, designated as *mdr4-2* (Figure 2A). Both mutations are predicted to result in premature termination of protein translation, and the alleles are therefore designated as predicted loss-of-function mutations (Figure S3).

Wild type and *mdr4* mutants were cultured in half-strength Kimura B medium with 5 μM CdCl_2_ to investigate the phenotype of *mdr4* mutants at seedling stage. Compared with wild-type plants, *mdr4* mutants exhibited a slight decrease in shoot length and dry weight, although no significant differences were observed in root growth (Figure 2B–D). Subsequently, we measured Cd, iron, zinc and manganese concentrations in roots and shoots of wild type and *mdr4* mutants. Cd concentration in *mdr4* mutants was reduced by 32.9–37.7% in shoots and 19.3–20.3% in roots compared to wild-type plants (Figure 2E,F). Additionally, Fe concentration in *mdr4* mutants was lower, with reductions of 14.3–18.4% in shoots and 27.7–33.6% in roots, suggesting a possible effect of OsMDR4 on Fe homeostasis, although direct Fe transport activity has not been demonstrated (Figure 2E,F). Zn and Mn concentrations, however, remained comparable between wild type and *mdr4* mutants (Figure 2E,F).

### 2.3. OsMDR4 Is Involved in Cd Accumulation in Rice Roots

**Figure 3:**
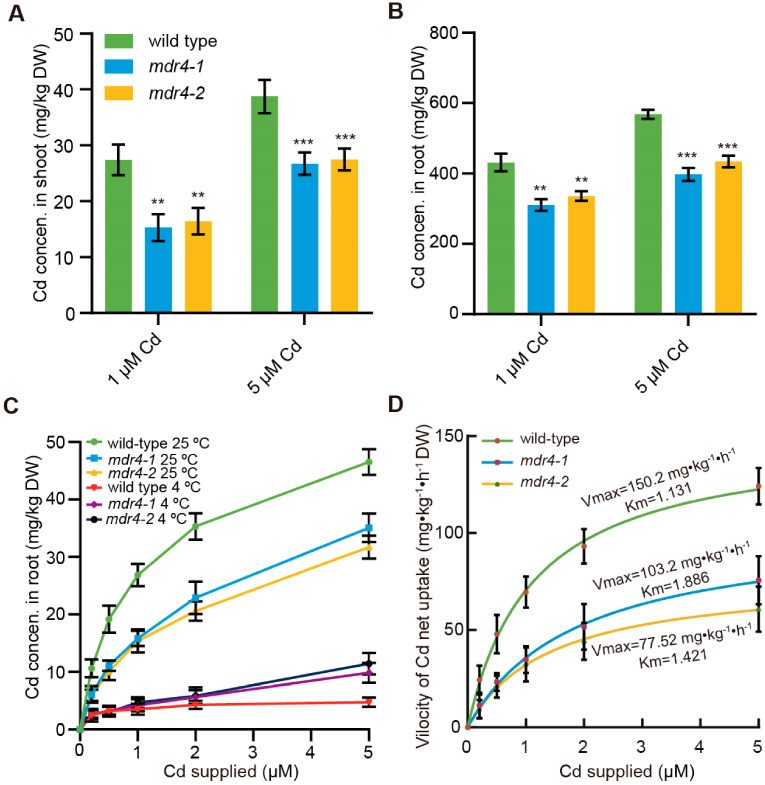
Cadmium uptake kinetics in wild-type and *mdr4* mutant seedlings. (**A**,**B**) Cd concentrations in shoots (**A**) and roots (**B**) of wild-type and *mdr4* mutants exposed to 1 or 5 µM Cd for 14 days. (**C**) Root-associated Cd in wild-type and *mdr4* mutants measured at 25 °C and 4 °C after 20 min of exposure to varying external Cd concentrations. (**D**) Net Cd uptake (25 °C minus 4 °C) in wild-type and *mdr4* mutants as a function of external Cd concentration. Data were fitted to the Michaelis–Menten plot method. Values represent means ± SD from three independent biological replicates (n = 3). Statistical significance was determined by Student’s *t*-test (** *p* < 0.01, *** *p* < 0.001).

To further investigate the contribution of OsMDR4 to Cd absorption in rice, wild type and *mdr4* mutants were treated with 1 or 5 μM CdCl_2_ for two weeks, and the Cd concentrations in roots and shoots were measured. Compared to wild-type plants, Cd concentration in shoots of *mdr4* mutants decreased by 40.6–44.2% at 1 μM CdCl_2_ and by 29.1–31.1% at 5 μM CdCl_2_ (Figure 3A). Similarly, Cd concentration in the roots of *mdr4* mutants decreased by 21.3–36.5% at 1 μM CdCl_2_ and by 23.6–30.8% at 5 μM CdCl_2_ (Figure 3B).

A Cd uptake kinetics assay was conducted to further evaluate the role of OsMDR4 in Cd absorption. Cd accumulation in the roots of wild-type plants was consistently higher than in the two *mdr4* mutants across Cd concentrations of 0.2, 0.5, 1.0, 2.0 and 5.0 μM (Figure 3C). Cd content in roots at low temperature (4 °C) was attributed to apoplastic Cd adsorption [27,28,29]. To analyze the symplastic Cd absorption, net Cd uptake was calculated by subtracting absorption at 4 °C from that at 25 °C, and the data were fitted to the Michaelis–Menten equation using nonlinear regression (95% confidence intervals, R^2^ = 0.9740, 0.9321 and 0.9134 for wild-type and the two *mdr4* mutants, respectively). The Km values of *mdr4* mutants were higher than those of wild-type plants, and the Vmax values of the two mutants were 31.29–48.39% lower than those of wild-type plants (Figure 3D).

**Figure 4:**
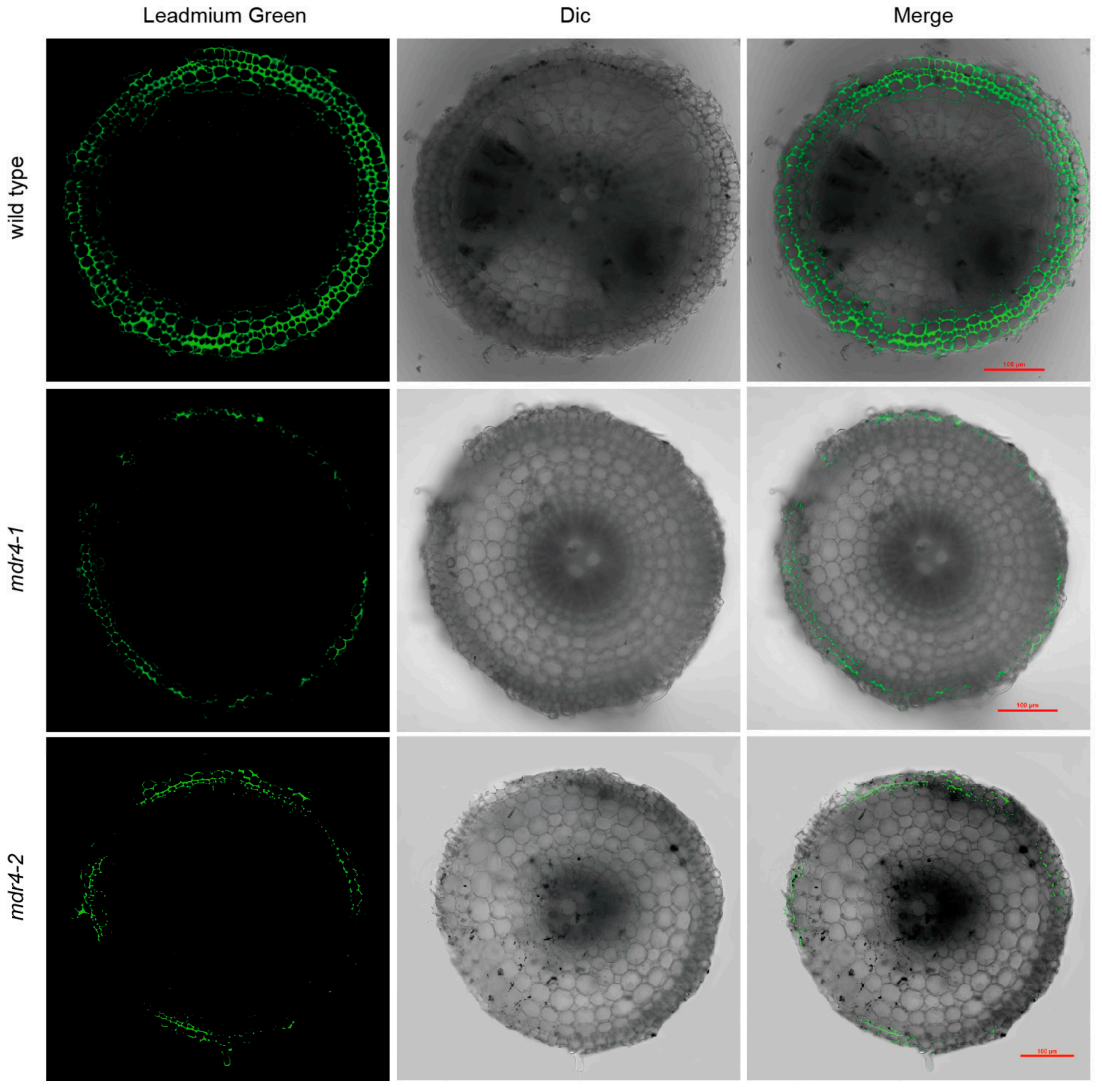
The cadmium distribution in wild type or *mdr4* mutants root. The cadmium probe was laser-excited at 488 nm, and the emitted fluorescence signaling was collected with a band-pass filter at 490–540 nm (GFP panel). Fluorescence images shown are representative of at least three independent biological replicates (n = 3).

Additionally, a green fluorescence probe was used to label Cd in the roots of wild-type and *mdr4* mutants at the three-leaf stage after treatment with 0.5 µM CdCl_2_ for three days. The green fluorescent signal observed in the epidermis of wild-type roots was visually stronger than that in *mdr4* mutants, suggesting reduced Cd-associated fluorescence in the mutant roots (Figure 4). However, this staining is qualitative and should not be interpreted as quantitative proof of Cd transport activity.

Together, these data indicate that OsMDR4 contributes to cellular Cd accumulation.

### 2.4. OsMDR4 Is Expressed in Roots and Induced by Cd Supply

**Figure 5:**
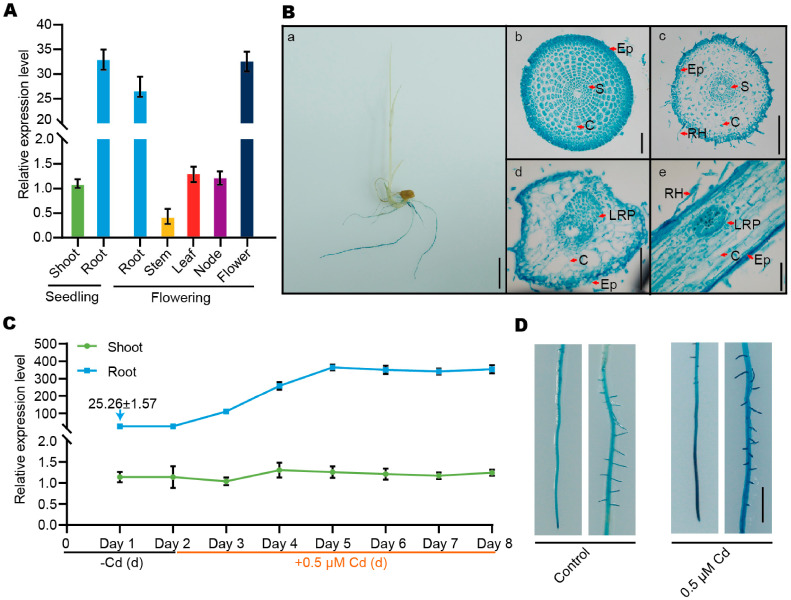
The expression pattern of *OsMDR4*. (**A**) The q-PCR analysis of *OsMDR4* transcript levels in diverse tissues. Data are average values of three independent experiments and are presented as mean ± SD. (**B**) Histochemical GUS staining of *OsMDR4* pro:GUS plants. (**a**) Seedling stage, scale bar = 1 cm. (**b**–**e**) Paraffin section of root, scale bar = 1 mm. (**b**) Cross slice of meristematic zone. (**c**) Cross slice of mature zone. (**d**) Cross slice of lateral root primordium. (**e**) Longitudinal slice of lateral root primordium. (**C**) Induction of *OsMDR4* expression level in root and shoot treated with 0.5 μM Cd for different times. (**D**) Histochemical GUS staining of OsMDR4 pro:GUS root under 0 or 0.5 μM Cd condition for 3 days, scale bar = 1 mm. Three independent transgenic lines carrying the OsMDR4Pro::GUS construct were examined, and consistent staining patterns were observed across all lines. Representative images from one line are shown.

Quantitative real-time PCR (qRT-PCR) was used to investigate the expression pattern of OsMDR4 in different tissues of Nipponbare grown in Kimura B hydroponic at various stages. At the seedling stage, OsMDR4 was strongly expressed in roots, with expression levels approximately 30-fold higher than in shoots (Figure 5A). At the flowering stage, the relative expression level in roots decreased by 17.0%, while high expression was detected in flowers (Figure 5A).

To analyze tissue-specific expression in detail, transgenic plants carrying the OsMDR4Pro::GUS were generated. Consistent with the qRT-PCR results, high GUS activity was detected in roots at the seedling stage and in floral organs at the flowering stage (Figure 5B(a) and Figure S4A,B). GUS signals were observed throughout the cross-section of the meristematic zone of roots (Figure 5B(b)), whereas weaker signals were detected in the cortex at the maturation zone (Figure 5B(c)). OsMDR4 expression was also prominent in root hairs and lateral root primordia (Figure 5B(d,e)). Pollen grains and connective vascular bundles of anther showed strong staining (Figure S4C,D).

To confirm Cd-induced expression, 3-week-old wild-type (Nipponbare) seedlings were exposed to 0.5 μM Cd for 6 days. qRT-PCR revealed that Cd exposure significantly increased OsMDR4 expression level in roots but not in shoots, with an approximately peak 13-fold increase on day three (Figure 5C). GUS activity mirrored this trend, with increased signals in the meristematic and mature zones following Cd treatment (Figure 5D).

In summary, OsMDR4 is primarily expressed in rice roots and flowers, and its expression is significantly induced by Cd exposure in roots.

### 2.5. OsMDR4 Is Localized to Plasma Membrane

Subcellular localization is key to understanding the function of a transporter. To determine the subcellular localization of OsMDR4, an enhanced green fluorescent protein (EGFP) reporter construct was generated to express a fusion protein with EGFP fused to the C-terminus of OsMDR4. Expression of the fusion protein was driven by a cauliflower mosaic virus 35S promoter.

**Figure 6:**
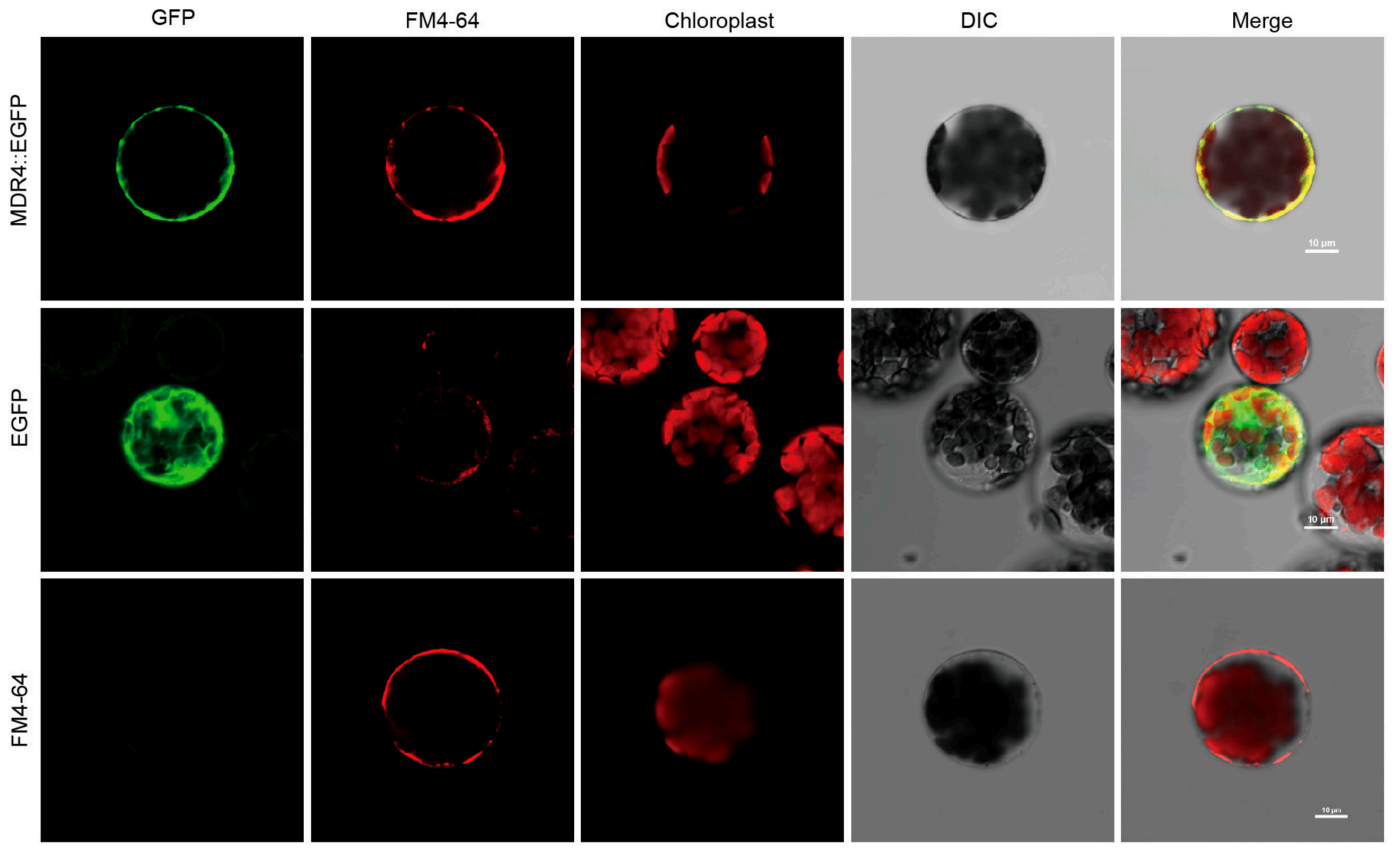
Subcellular localization of OsMDR4 in protoplasts from Arabidopsis mesophyll cells. The recombinant plasmid 35S::OsMDR4–EGFP was used to transfer into Arabidopsis mesophyll protoplasts transiently, and staining with plasma membrane marker FM4-64. OsMDR4–EGFP fusion protein was laser-excited at 488 nm, and the emitted fluorescence signaling was collected with a band-pass filter at 490–540 nm (GFP panel). FM4-64 marker was laser-excited at 561 nm, and the emitted fluorescence was collected with a band-pass filter at 592 nm (RFP panel). The GFP and RFP fluorescence signals were mostly overlapping (overlay panel). The DIC panel shows the bright-field fluorescence microscopic image of the protoplast, scale bar = 10 µm.

To analyze the subcellular localization of OsMDR4, we transiently expressed OsMDR4–EGFP and EGFP alone in Arabidopsis mesophyll protoplasts. The plasma membrane marker FM4-64 (Thermo Fisher Scientific) was used for co-localization. The red fluorescent signal produced by FM4-64 showed apparent co-localization with the green fluorescent signal generated by OsMDR4–EGFP. Cells transformed with the EGFP empty vector exhibited EGFP signals throughout the cell (Figure 6 and Figure S5). No EGFP signal was detected in the mesophyll protoplasts without plasmid transformation (Figure 6).

In conclusion, these results confirm that OsMDR4 is localized to the plasma membrane of cells.

## 3. Discussion

A growing body of evidence has established that Cd accumulation in rice is governed by a complex network of transporters with distinct subcellular localizations, tissue specificities, and transport directions. Among the well-characterized players, OsNRAMP5 functions as a plasma membrane-localized influx transporter responsible for the major uptake of Cd, as well as Mn and Fe, into root cells [9,10,11]. In contrast, OsHMA3 sequesters Cd into root vacuoles, thereby limiting its translocation to shoots [30], whereas OsLCT1 mediates Cd loading into the phloem and contributes to Cd distribution to the grain [15]. Members of the OsZIP family, such as OsZIP7, facilitate Zn and Cd delivery to the grain through xylem loading and vascular transfer [31], and several OsABCG transporters have been implicated in Cd efflux or detoxification [32,33].

In this context, OsMDR4 exhibits a functional profile that distinguishes it from these previously characterized transporters. Unlike OsNRAMP5, which functions as a major Cd uptake system in roots and also transports Mn and Fe, OsMDR4 contributes to Cd accumulation with a distinct expression pattern that includes high transcript levels in both roots and floral organs (Figure 5A). Unlike the vacuolar sequestration function of OsHMA3, which reduces Cd mobility, OsMDR4 is localized to the plasma membrane (Figure 6), consistent with a role at the entry point of Cd into root cells. Unlike OsABCG transporters, which typically function as efflux transporters, the *mdr4* mutant phenotypes suggest that OsMDR4 contributes to Cd accumulation rather than Cd exclusion. Collectively, these comparisons place OsMDR4 as a previously uncharacterized plasma membrane-localized protein that contributes to Cd accumulation in rice, with a distinctive expression profile and mutant phenotype that distinguish it from other known Cd transporters. Further mechanistic studies will be required to determine the precise transport mode and substrate selectivity of OsMDR4 relative to these transporters.

Notably, many heavy metal transporters exhibit broad substrate specificity, transporting both toxic metals and essential micronutrients. In rice, OsNRAMP5 functions as a major transporter for Cd, Mn, and Fe uptake into roots [9,10,11,30]; OsIRT1 and OsIRT2 mediate Fe and Cd transport [12,34]; and OsZIP family members are involved in Zn, Fe, and Cd transport [31,35,36,37,38]. This functional promiscuity raises the important question of whether OsMDR4, in addition to its role in Cd accumulation, may also affect the homeostasis of essential mineral nutrients.

**Figure 7:**
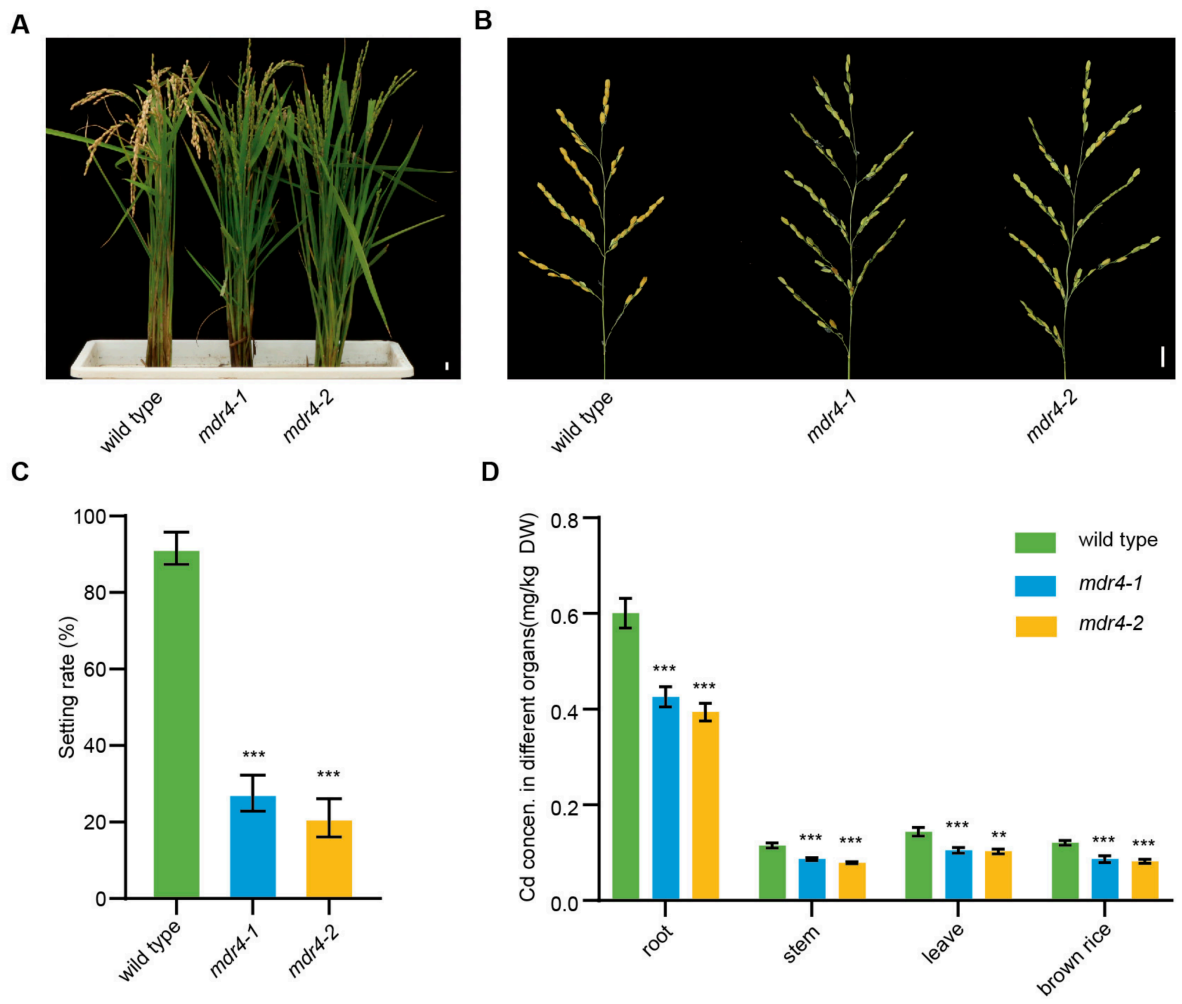
The setting rate and cadmium concentration of wild type and *mdr4* mutants. (**A**) Morphological comparison of wild type and *mdr4* mutants at maturity. (**B**) Morphological comparison of wild type and *mdr4* mutants spike. (**C**) The setting rate of wild type and *mdr4* mutants. (**D**) Cadmium concentration in different organs of wild type and *mdr4* mutants. Seed-setting rate (%) = (number of filled grains/total spikelets) × 100. For each biological replicate, 10 plants were analyzed, with 3 panicles per plant. Data represent means ± SD from three independent biological replicates. Values represent means ± SD (n = 3) from three biological replicates. Statistical significance of the difference between the wild type and *mdr4* mutants was determined by a *t*-test (** *p* < 0.01; *** *p* < 0.001).

Our data show that *mdr4* mutants exhibited reduced Fe concentrations in both roots and shoots compared with wild-type plants (Figure 2E,F), suggesting a possible link between OsMDR4 function and Fe homeostasis. However, it must be emphasized that our current data do not demonstrate direct Fe transport by OsMDR4. The observed alterations in Fe levels could result from indirect effects, such as changes in root physiology, altered expression of other metal transporters, or pleiotropic effects of the mutation on plant development. Alternatively, Cd and Fe may compete for shared transport pathways, and reduced Cd uptake in *mdr4* mutants might indirectly affect Fe distribution. The reduced grain-setting rate observed in *mdr4* mutants (Figure 7A–C) could also reflect broader physiological disturbances rather than specific metal transport defects, and complementation experiments would be required to confirm that these phenotypes are directly attributable to loss of OsMDR4 function.

Given these uncertainties, we cannot conclude that OsMDR4 directly transports Fe or other essential metals. Nevertheless, the altered Fe accumulation phenotype warrants further investigation. Future studies should examine whether OsMDR4 affects Zn, Mn, or Cu homeostasis and whether it functions as a transporter for these metals or indirectly modulates their distribution through regulatory mechanisms.

Beyond substrate specificity, the interpretation of our uptake kinetic data also warrants consideration of methodological limitations. The uptake measured at 4 °C was subtracted from that at 25 °C to estimate symplastic Cd influx, as described previously [28]. This approach is based on the assumption that active transport is largely suppressed at low temperature, whereas apoplastic binding and passive diffusion are less affected. However, it should be noted that this assumption has inherent limitations. Low temperature can influence membrane fluidity, protein conformation, and non-specific binding, potentially affecting both apoplastic and symplastic fractions. Additionally, some active transport processes may not be completely abolished at 4 °C, and the extent to which metabolism is suppressed can vary depending on the plant species, tissue type, and exposure duration. Consequently, the subtracted net uptake values should be interpreted as approximations of symplastic influx rather than absolute measurements. To minimize surface-bound Cd, we used a washing step with Cd-free uptake solution prior to digestion; nonetheless, we acknowledge that this may not fully remove all apoplastically bound Cd.

We acknowledge that the reduced shoot Cd accumulation in *mdr4* mutants is consistent with reduced root uptake, but we cannot rule out additional effects on xylem loading or root-to-shoot translocation. Future studies measuring xylem sap Cd concentrations would help distinguish between these possibilities.

The tissue-specific expression pattern of OsMDR4 further supports its physiological role in Cd uptake. As sessile organisms, plants cannot move to obtain nutrients, and the root serves as a crucial organ for nutrient absorption in rice. A variety of key transporters involved in nutrient uptake are abundantly expressed in the root, each exhibiting distinct expression patterns. OsNRAMP5 is primarily expressed in the exodermis and endodermis of the root mature region and is involved in manganese and Cd absorption from the external solution into the cytoplasm [11]. OsIRT1 functions as an iron and Cd transporter in rice; GUS analysis demonstrated that OsIRT1 is expressed in the epidermis and exodermis of the elongating zone, as well as in the inner layer of the cortex in the mature zone of Fe-deficient roots [12]. OsAMT1.1 is expressed in root epidermis and stele parenchyma, where it plays a role in ammonium uptake and ammonium–potassium homeostasis across a range of low and high ammonium concentrations [39].

Our results show that OsMDR4 exhibits high expression in the root epidermis, stele, and root hairs (Figure 5B), all of which are critical structures for nutrient uptake. Knockout of OsMDR4 resulted in reduced cadmium content within the root (Figure 3B and Figure 4), suggesting that OsMDR4 contributes to cadmium accumulation in rice roots.

The high expression of *OsMDR4* in floral organs (Figure 5A and Figure S4C,D) suggests a possible role in reproductive development. One possibility is that OsMDR4 contributes to Cd delivery to developing seeds, thereby affecting grain Cd accumulation. Alternatively, it may play a role in maintaining metal homeostasis during reproduction. This is consistent with the reduced seed-setting rate observed in *mdr4* mutants (Figure 7A–C). However, these interpretations remain speculative, and future studies are needed to elucidate the significance of floral *OsMDR4* expression. We also acknowledge that the reduced seed-setting rate is a pleiotropic effect that could independently reduce grain Cd accumulation through decreased sink strength, complicating the interpretation of the mutant phenotype.

The functional implications of these expression patterns are further informed by considering the broader context of ABC transporter functions. ABC transporters are frequently involved in the detoxification of various xenobiotics. AtMRP6 is primarily expressed in the apical meristem and is induced by Cd treatment [40]. AtMRP3 functions as a transporter of phytochelatin–Cd(PC–Cd) complexes, with its activity regulated by Cd and coordinated with AtMRP1 and AtMRP2 [41]. Overexpression of AtPDR8 enhances plant resistance to Cd and Pb [19]. OsABCG36/OsPDR9 is localized to the plasma membrane and plays a role in Cd detoxification [33]. Additionally, OsPDR20 is strongly induced in roots and shoots of seedlings under Cd stress, and its knockdown leads to increased Cd accumulation in plants [32].

It is well established that many plant ABC transporters do not transport free metal ions directly but rather function as transporters of metal–ligand complexes. In yeast and plants, cadmium can form complexes with glutathione or phytochelatins, and these conjugates are subsequently transported into vacuoles or out of cells via ABC transporters [21,42]. For instance, the rice ABC transporter OsABCG36/OsPDR9 functions as an efflux transporter for Cd or Cd conjugates at the plasma membrane [33], whereas OsABCC9 mediates Cd tolerance by sequestering Cd into root vacuoles, likely in a complexed form [43]. Moreover, structural studies on human ABCB6 have revealed that Cd(II) binds to the transporter in coordination with GSH and PC2, providing direct molecular evidence for the recognition of Cd–thiol peptide complexes by ABC transporters [44]. In Arabidopsis, ABCC-type transporters have been shown to transport phytochelatin–Cd complexes into vacuoles [20,41]. These precedents raise the important question of whether OsMDR4 transports free Cd^2+^ ions or Cd complexed with organic ligands. Given that OsMDR4 belongs to the ABC transporter family, which generally favors conjugated substrates, it is plausible that OsMDR4 may recognize Cd–GSH or Cd–PC complexes rather than free ions. However, our current data do not distinguish between these possibilities. Future studies employing membrane vesicle transport assays with defined substrates or heterologous expression systems capable of assessing ligand-dependent transport will be required to determine the substrate specificity of OsMDR4.

The functional characterization of OsMDR4 raises an important question regarding the transport directionality of MDR-type ABC proteins. While most plant MDR transporters have been traditionally characterized as efflux pumps involved in xenobiotic detoxification and hormone export, compelling evidence indicates that some members of this subfamily can function in the opposite direction. Notably, CjMDR1 from *Coptis japonica* was shown to transport the alkaloid berberine in an inward direction when expressed in *Xenopus* oocytes, and this uptake was ATP-dependent and inhibited by classic ABC transporter inhibitors [45]. This finding was considered unexpected at the time because no eukaryotic ABC transporter had yet been reported to mediate substrate influx. Similarly, Arabidopsis ABCB transporters have been demonstrated to mediate both auxin and brassinosteroid transport through conformational changes between inward- and outward-facing states, suggesting that the transport directionality of MDR-type ABC proteins is not fixed but rather substrate- and context-dependent [46]. These precedents support the possibility that OsMDR4, as a plasma membrane-localized MDR-type protein, could contribute to cadmium accumulation by mediating inward transport. However, it must be emphasized that our current data do not distinguish between direct Cd transport by OsMDR4 and indirect regulation of other transport systems, and direct ATP-dependent transport activity remains to be demonstrated. Future studies employing ATPase assays, membrane vesicle transport experiments, or heterologous expression in *Xenopus* oocytes will be required to definitively establish the transport mechanism and directionality of OsMDR4.

In addition to metal transport, plant hormones are another class of substrates transported by ABC transporters in plants. Beyond AtMDR1, AtMDR4, AtMDR11, and MDR17, which are involved in auxin transport, AtPDR8 promotes the efflux of auxin precursor indole-3-butyric acid (IBA), thereby regulating auxin homeostasis in plant cells [47]. AtPDR9, a homolog of AtPDR8, specifically facilitates the efflux of 2,4-D from plant cells without affecting endogenous auxin transport [48].

The coleoptile is a crucial organ for auxin production and transport in gramineous plants [49]. The strong GUS staining observed in the coleoptile raises the possibility that OsMDR4 may be involved in auxin-related processes (Figure S4A,B). However, it must be noted that GUS staining in the coleoptile does not constitute direct evidence for auxin transport, and this interpretation remains speculative in the absence of auxin-specific transport assays.

Finally, subcellular localization provides important clues for transporter function analysis. OsVIT1 and OsVIT2 are localized to the vacuolar membrane, where they mediate the translocation of iron (Fe) and zinc (Zn) between the cytoplasm and the vacuole. Both OsVIT1 and OsVIT2 partially rescued Fe^2+^ sensitivity in the *Δccc1* yeast mutant [50]. OsMIT is localized to the mitochondria and complemented the growth of *Δmrs3Δmrs4* yeast mutants defective in mitochondrial Fe transport [51]. OsNRAMP1, localized to the plasma membrane, is able to complement the *Δfet3fet4* yeast mutant [28,52].

In this study, we demonstrated that OsMDR4 is localized to the plasma membrane (Figure 6). Heterologous expression of OsMDR4 increased Cd^2+^ sensitivity in the *Δycf1* yeast mutant (Figure 1), suggesting that OsMDR4 contributes to cadmium accumulation. We acknowledge that heterologous expression in yeast cannot fully reproduce the native membrane environment or regulatory context of rice root cells and that the observed yeast phenotype may result from indirect effects. Nevertheless, the *mdr4* mutant phenotypes provide more physiologically relevant evidence supporting a role for OsMDR4 in Cd accumulation in plants.

## 4. Materials and Methods

### 4.1. Plant Materials and Growth Conditions

Rice (*O. sativa* L. japonica cv Nipponbare) was used as the wild type. The transgenic plants were generated following established protocols [53]. *mdr4* homozygous knockout plants were confirmed by Sanger sequencing of the target locus. Potential off-target mutations were evaluated by sequencing the top five predicted off-target sites; no off-target mutations were detected in any of the lines used in this study. Rice seeds were rinsed in distilled water and incubated at 30 °C for two days to initiate germination. The germinated seeds were then transferred to hydroponic solutions and grown under controlled conditions, with a day/night temperature cycle of 30 °C for 14 h and 25 °C for 10 h. The plants were cultivated in half-strength Kimura B nutrient solution at pH 5.5 and modified according to published protocols. The composition of the solution was as follows: (NH_4_)_2_SO_4_ 24.1 mg/L, KNO_3_ 9.25 mg/L, K_2_SO_4_ 7.5 mg/L, MgSO_4_·7H_2_O 67.55 mg/L, KH_2_PO_4_ 12.4 mg/L, Ca(NO_3_)_2_·4H_2_O 43.2 mg/L, MnCl_2_·4H_2_O 0.905 mg/L, H_3_BO_3_ 1.43 mg/L, ZnSO_4_·7H_2_O 0.11 mg/L, CuSO_4_·5H_2_O 0.04 mg/L, H_2_MoO_4_·H_2_O 0.045 mg/L, and NaFeEDTA 3.67 mg/L. The nutrient solution was renewed every three days to maintain consistent metal availability [54]. Two-week-old rice seedlings were exposed to 1 or 5 µM CdCl_2_ for 14 days. The Cd-containing solutions were renewed every 3 days to maintain constant Cd concentrations. Each experiment was conducted at least three times with three biological replicates. For mature-plant experiments, rice plants were grown in paddy soil spiked with 5.0 mg kg^−1^ Cd. Plants were irrigated daily with tap water and fertilized with NPK at recommended rates. At maturity, panicles were harvested when 90% of grains turned yellow. Growth conditions were 28 °C/22 °C day/night, 14 h photoperiod, and 70% relative humidity.

### 4.2. Plasmid Construction

For heterologous expression in yeast, the full-length OsMDR4 coding sequence (CDS) was amplified from wild-type cDNA using specific primers containing overlapping sequences with the *pYES2* vector (Table S1). The amplified products were recombined into the BamH I- and Xba I-digested pYES2 using the ConeExpress II one step cloning Kit (Vazyme, Nanjing, China).

To generate *mdr4* mutants, two target sequences from the OsMDR4 coding region were selected for primer design, incorporating Bsa I restriction sites (Table S1). The primers were annealed to form a spacer, which were then cloned into the pOs-sgRNA vector using T4 ligase (Thermo Fisher Scientific, Shanghai, China). The constructed pOs-sgRNA vector was subsequently recombined with the UbiPro::Cas9 vector using LR Clonase II enzyme Mix (Thermo Fisher Scientific, Shanghai, China) [55].

For the OsMDR4 expression pattern assay, a 2000 bp DNA sequence upstream of the OsMDR4 start code (ATG) was amplified from wild-type genomic DNA using the specific primers containing overlapping sequences for the pCAMBIA1301 vector (Table S1). The amplified fragment was then recombined into the EcoR I- and Hind III-digested pCAMBIA1301 vector using the ClonExpress II one step cloning Kit (Vazyme).

To analyze the subcellular localization of OsMDR4, the coding sequence of OsMDR4, excluding stop codon, was amplified from wild-type cDNA using specific primers with overlapping sequence for the pCAMBIA2300-EGFP vector (Table S1). The amplified fragments were then recombined into the BamH I- and Xba I-digested pCAMBIA2300-EGFP vector using the ClonExpress II one step cloning Kit (Vazyme).

### 4.3. Heterologous Expression in Yeast

The expression vector, either a vector control or recombinant construct, was transferred into a Cd sensitive yeast strain *Δycf1* (MATa; *his3Δ1*; *leu2Δ0*; *met15Δ0*; *ura3Δ0*; YDR135c::kanMX4) using the PEG/LiAc-mediated method [56]. Transformed yeast cells were selected on an SD medium lacking uracil (SD-Ura), and positive clones were confirmed by yeast colony PCR. These verified clones were cultured in SD-Ura liquid medium until reaching the early log phase for subsequent growth assays.

For the spot assay, five microliters of cell suspension at an OD600 value of 0.5 and four serial 1:10 dilutions were spotted onto SD-Ura plates (containing 2% D-glucose) or SG-Ura plates (containing 2% galactose), with or without 20 μM CdCl_2_. Plates were incubated at 30 °C for three days, and growth was compared to assess the effects of Cd treatment.

For liquid growth assays, yeast cells were inoculated at an OD600 of 0.05 in a 96-well plate (200 µL per well) and grown at 30 °C with shaking at 200 rpm. OD600 readings were taken every 2 h for 48 h using a microplate reader. For intracellular Cd measurements, yeast cells were grown to an OD600 of 0.5, induced with 2% galactose for 6 h, and then treated with 10 µM Cd for 24 h. Cells were harvested and processed for Cd content measurement.

### 4.4. Determination of Metals Concentration

For plant samples, different rice tissues were first dried in an oven at 105 °C for 12 h, followed by drying at 70 °C for 3 days to constant weight. The dried samples were then weighed and digested using a HNO_3_/HClO_4_ (*v*/*v* = 17:3) solution in a heating block at 160–180 °C. For yeast samples, cells were washed three times with ice-cold 10 mM EDTA (pH 8.0) and twice with ice-cold distilled water to remove surface-bound metals and then pelleted and dried at 70 °C to constant weight. Cd concentrations were determined using ICP-MS (Agilent 7700x, Santa Clara, CA, USA) (Isotope monitored: ^111^Cd. Internal standard: ^115^In. Calibration range: 0.1–100 µg L^−1^). Certified reference material (NIST SRM 1568b rice flour, Gaithersburg, MD, USA) was used for quality control. Recovery was 95–105%. The detection limit was 0.01 µg L^−1^. Blanks were included in each digestion batch, and matrix effects were controlled by standard addition.

### 4.5. Visualization of Cd in Yeasts

Yeast strains were cultured in SG medium with 20 μM CdCl_2_ at 30 °C for 24 h. Cells were harvested by centrifugation at 6000 rpm for 5 min and washed five times with 0.85% NaCl. The washed yeast cells were resuspended in warm saline to a concentration of 1 × 10^6^ cells/mL. To each tube, 4 μL of LeadmiumGreen AM dye (ThermoFisher Scientific) was added; Leadmium™ Green AM dye was used at a final concentration of 5 µM. Cells were stained at 37 °C for 30 min, protected from light. Fluorescence signals were detected and analyzed with a Nikon, Ti-E confocal microscope. Excitation was performed with a 488 nm laser, and the emitted fluorescence was collected using a band-pass filter in the range of 490–540 nm; laser power and detector gain were optimized to avoid saturation and minimize photobleaching. Each experiment was conducted at least three times with three biological replicates.

### 4.6. Visualization of Cd in Rice Roots

Rice plants were treated with a solution containing 0.5 μM CdCl_2_ for 3 days. Treated roots of seedlings were washed with 0.85% NaCl solution 3 times, 1 min each time; cut; and stained with Leadmium™ Green AM dye (ThermoFisher Scientific) for 30 min. The free probe was washed with 0.85% NaCl solution, protected from light. Root sections were taken from the elongation zone (approximately 5–15 mm from the root tip) for fluorescence analysis. Fluorescence was detected and analyzed with a Nikon, Ti-E confocal microscope. Fluorescence was excited by a laser at 488 nm, and the emitted fluorescence was collected with a band-pass filter at 490–540 nm; laser power and detector gain were optimized to avoid saturation and minimize photobleaching. Each experiment was conducted at least three times with three biological replicates.

### 4.7. Cd Influx Kinetics Assay

The Cd influx kinetics assay was performed according to a previously published report [28]. Three-week-old seedlings of wild type and two *mdr4* mutants were pre-treated in the uptake solution (0.5 mM CaCl_2_, 2 mM MES, pH 5.6) at room temperature for 12 h and then transferred into uptake solutions containing Cd concentrations of 0, 0.2, 0.5, 1.0, 2.0 and 5.0 μM at 4 °C and 25 °C for 20 min under continuous aeration. After Cd exposure, roots were first rinsed with Cd-free uptake solution, then immersed in ice-cold 5 mM CaCl_2_ for 10 min to remove apoplastically bound Cd, and finally rinsed twice with deionized water. Root tissue was dried at 65 °C for 3 days before determination of Cd concentration. Uptake velocity (V) was calculated as V = (Cd_root/DW_root)/t, where Cd_root is the Cd content in roots (µg), DW_root is root dry weight (g), and t is the incubation time. Values are expressed as nmol g^−1^ FW h^−1^. Each experiment was conducted at least three times with three biological replicates.

### 4.8. Gene Expression Analysis

Rice tissues were collected, and total RNA was extracted using the Trizol reagent (Invitrogen, Shanghai, China). The PrimeScript™RT reagent Kit with gDNA Eraser (Perfect Real Time) (Takara, Beijing, China) was used to synthesize first-strand cDNA. Quantitative RT-PCR was performed in a QuantStudio 5 real-time PCR system (Thermo Fisher Scientific) using a Powerup SYBR Green Master Mix (Thermo Fisher Scientific) in a 10 μL volume. OsActin1 was used as an internal standard. Relative expression was calculated using the 2^−ΔΔCt^ method, with OsACTIN1 as the internal reference gene and wild-type root samples as the calibrator. Primer efficiencies were between 95% and 105%. Amplification specificity was confirmed by melt-curve analysis and agarose gel electrophoresis. The primers used for quantitative real-time PCR are listed in Table S1. Each experiment was conducted at least three times with three biological replicates.

### 4.9. Histochemical GUS Staining

Transgenic plants carrying the GUS reporter driven by the OsMDR4 promoter were selected based on hygromycin resistance. OsMDR4 promoter activity was visualized in different tissues of plants via histochemical staining of GUS. OsMDR4pro::GUS transgenic plants were immersed in staining buffer containing 0.1 M phosphate-buffered sodium (pH 7.0), 10 mM EDTA, 2 mM potassium ferrocyanide, 0.1% (*v*/*v*) Triton X-100, 20% (*v*/*v*) methanol, and 500 μM X-gluc. GUS staining was performed for 6–8 h at 37 °C. Negative controls using non-transgenic plants were included and showed no staining. The duration was chosen based on preliminary time-course experiments [57]. Tissues after staining were transferred into 75% alcohol to decolor and then examined and photographed with an Olympus SZX12 microscope (Olympus, Tokyo, Janpan) equipped with a camera or digital camera.

### 4.10. Paraffin Sections of Rice Root

The stained root samples were washed with 70% alcohol three times, two hours each time. Samples were gradually dehydrated in an increasing alcohol concentration gradient up to 100%, two hours for each alcohol concentration. Samples were transferred into 50% xylene and 50% ethanol solution for overnight, and we continued to increase the xylene proportion until 100% xylene. Next, the wax immersion of samples was performed in 50% xylene and 50% paraffin at 42 °C overnight and was continued at 60 °C for 4 h in an incubator. Subsequently, samples were transferred into 100% paraffin at 60 °C for 8 h, and the paraffin was renewed every 2 h. Finally, the samples were embedded in paraffin and sectioned using a Leica RM2015 rotary microtome (Leica, Beijing, China) [58].

### 4.11. Subcellular Localization

Arabidopsis mesophyll protoplasts were prepared from 30-day-old rosette leaf tissues by digesting leaves and shoot slices with 1.5% cellulase RS (Yakult, Tokyo, Japan) and 3% macerozyme RS (Yakult, Japan). The protoplasts were resuspended with W5 medium (154 mM NaCl, 125 mM CaCl_2_, 5 mM KCl, 2 mM MES, pH 5.7). A total of 10 μg OsMDR4-pCAMBIA2300-EGFP and pCAMBIA2300-EGFP plasmids were separately transfected into protoplasts by the PEG-mediated method [59]. The transformed protoplasts were incubated in the dark at 25 °C for 12–16 h.

The dye FM4-64 (a plasma membrane fluorescent marker) was used to stain the plasma membrane of protoplasts. FM4-64 staining was performed at a concentration of 10 µg/mL for 10 min, followed by a 5 min washing step. Imaging was performed immediately after washing to ensure plasma membrane-specific labeling. Fluorescence emitted by EGFP and FM4-64 was detected and analyzed with a Nikon, Ti-E confocal microscope (Nikon, Tokyo, Japan). EGFP protein is excited by a laser at 488 nm, and the emitted fluorescence is collected with a band-pass filter at 490–540 nm. FM4-64 is excited by a laser at 561 nm, and the emitted fluorescence is collected with a band-pass filter at 580–650 nm; laser power and detector gain were optimized to avoid saturation and minimize photobleaching.

### 4.12. Statistical Analysis

All experiments were performed with at least three independent biological replicates, and each replicate contained three technical replicates unless otherwise stated. All data are presented as means ± standard deviation. The normality of data distribution was assessed using the Shapiro–Wilk test, and homogeneity of variances was assessed using Levene’s test. For comparisons between two groups, Student’s *t*-test was applied. For comparisons involving more than two groups, one-way or two-way analysis of variance (ANOVA) was performed, followed by Tukey’s HSD (honestly significant difference) post hoc test for multiple comparisons. Kinetic parameters (Km and Vmax) were obtained by nonlinear regression fitting to the Michaelis–Menten equation using GraphPad Prism version 8.0 (GraphPad Software, San Diego, CA, USA). Statistical significance was set at *p* < 0.05. All statistical analyses were performed using SPSS Statistics version 20.0 (IBM, Armonk, NY, USA).

## 5. Conclusions

In summary, this study identifies and characterizes OsMDR4 as a previously uncharacterized plasma membrane-localized protein that contributes to cadmium accumulation in rice, with functional evidence from both plant and heterologous systems. However, we note that the severe reproductive defects observed in the *mdr4* mutants, including a markedly reduced seed-setting rate, preclude the direct application of OsMDR4 disruption as a straightforward strategy for breeding low-cadmium rice. Nevertheless, our findings provide a foundation for future research aimed at elucidating the role of OsMDR4 in the rice Cd transport network. Future work is needed to define the transport specificity of OsMDR4 toward other divalent metals and to elucidate its functional relationship with known Cd transporters, such as OsNRAMP5 and OsIRT, which will further clarify its role in the rice Cd transport network. Additionally, approaches such as tissue-specific or conditional knockdown strategies could be explored to minimize pleiotropic effects, with the goal of separating grain Cd reduction from yield penalties.

## Data Availability

The original contributions presented in this study are included in the article/Supplementary Materials. Further inquiries can be directed to the corresponding authors.

## Supplementary Materials

The following supporting information can be downloaded at: https://www.mdpi.com/article/10.3390/plants15152378/s1, Figure S1: Distribution of cadmium in yeast expressing OsMDR4, OsNRAMP5, or vector control; Figure S2: Predicted transmembrane topology of OsMDR4; Figure S3: Sequence alignment of OsMDR4 mutations and predicted protein truncations in *mdr4-1* and *mdr4-2* mutants; Figure S4: Tissue-specific GUS expression analysis of OsMDR4 promoter activity in rice anthers and coleoptiles; Figure S5: Line-scan fluorescence intensity analysis of OsMDR4–EGFP co-localization with the plasma membrane marker FM4-64; Table S1: Primers used in this study.

## Abbreviations

The following abbreviations are used in this manuscript:

GFP	Green Fluorescent Protein
MDR	Multidrug Resistance Protein
PCR	Polymerase Chain Reaction
