# Supplementary material for: The Multidrug Resistance Protein OsMDR4 Is Involved in Cadmium Absorption in Rice (Oryza sativa L.)
Source: Plants (Basel). 2026 Aug 3;15(15):2378. doi: 10.3390/plants15152378 (PMC13468201; doi:10.3390/plants15152378)
Supplement: Supplementary file 1 [file plants-15-02378-s001.zip › Supplementary File-R.pdf]

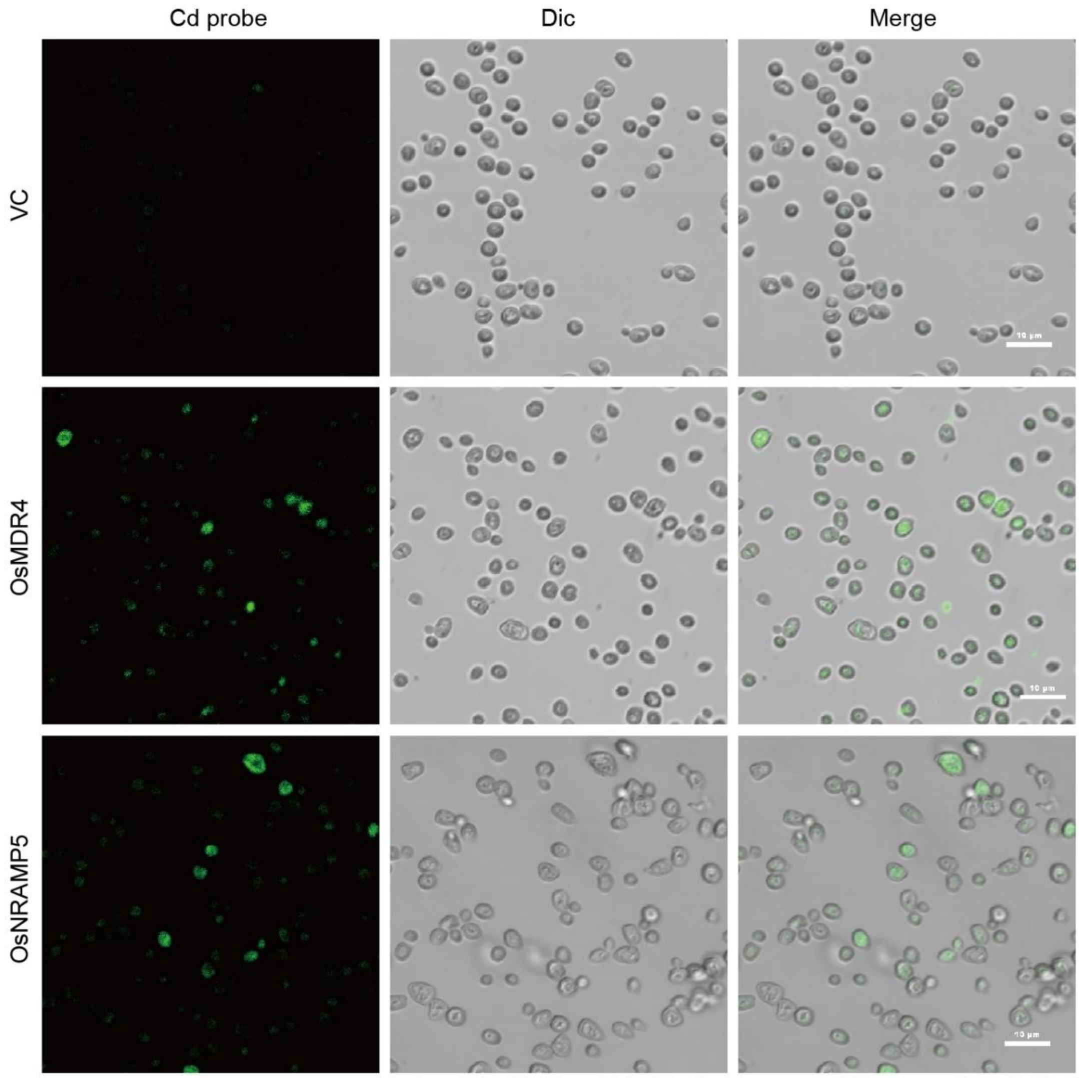

Figure S1. Distribution of cadmium in yeast expressing OsMDR4, OsNRAMP5, or vector control.

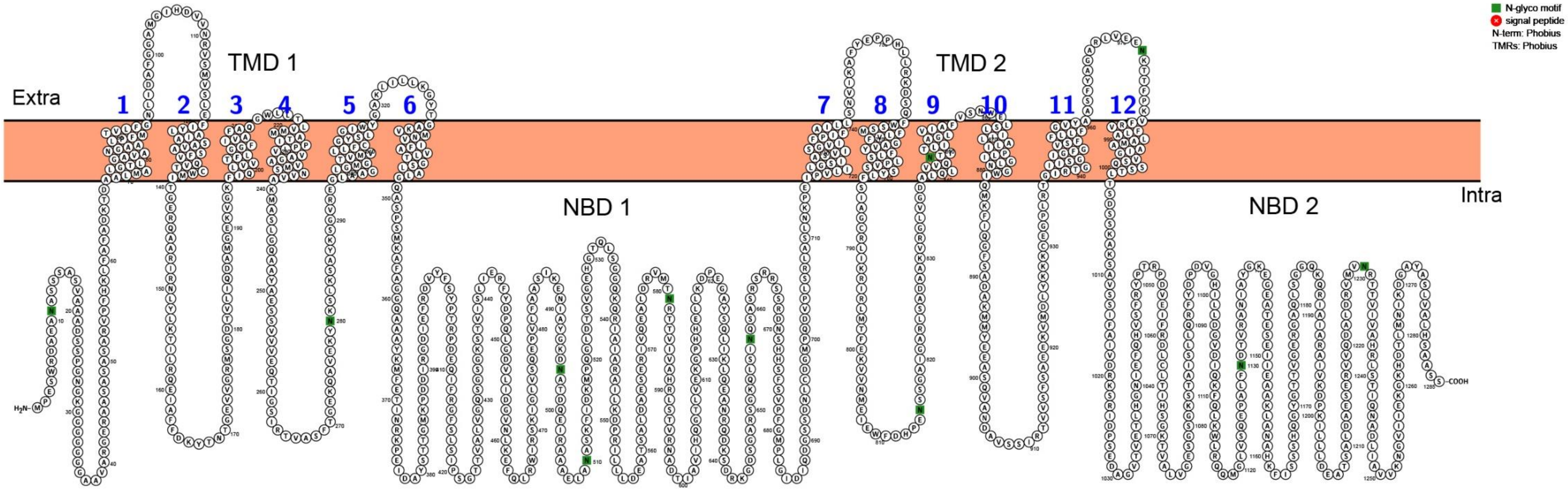

Figure S2. Predicted transmembrane topology of OsMDR4.

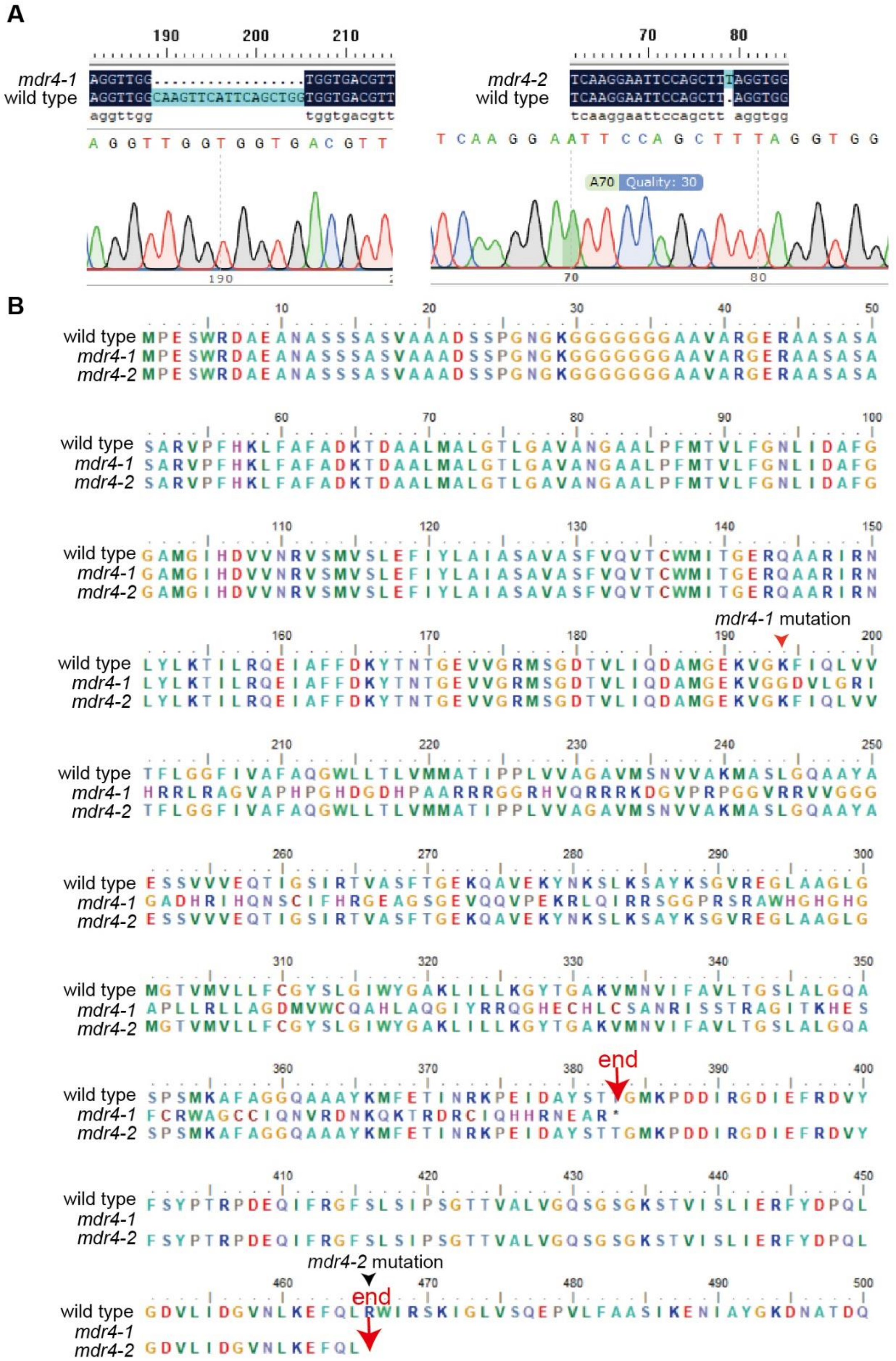

Figure S3. Sequence alignment of OsMDR4 mutations and predicted protein truncations in *mdr4-1* and *mdr4-2* mutants.

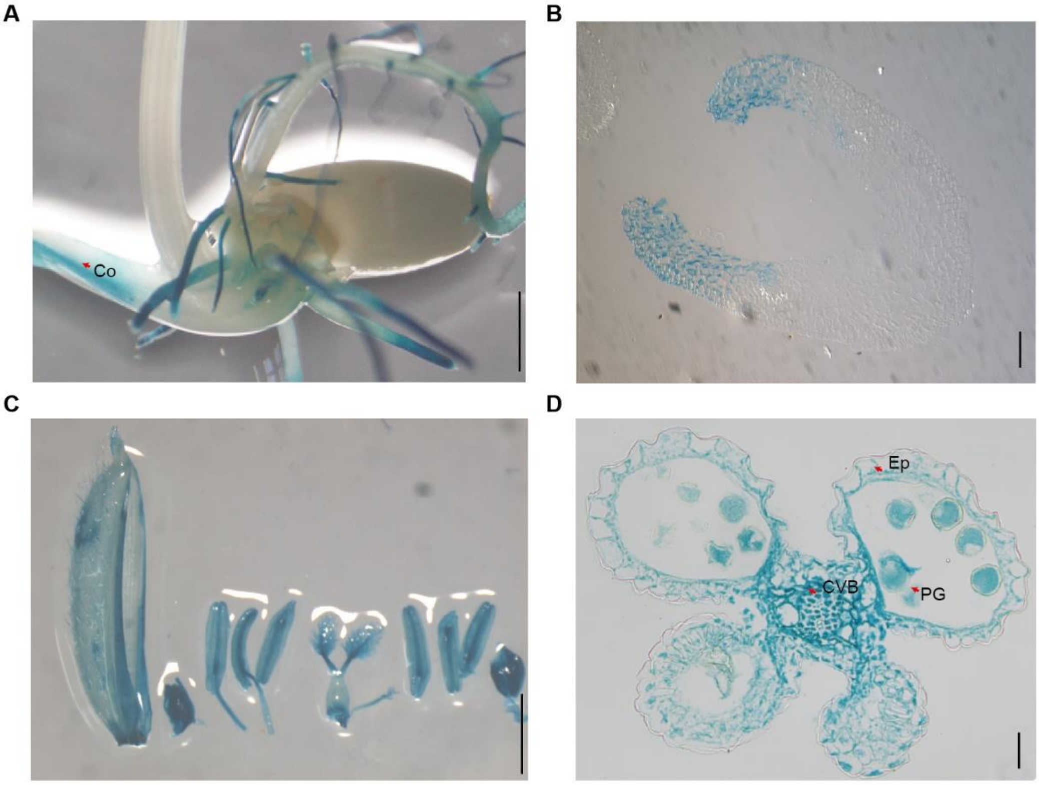

Figure S4. Tissue-specific GUS expression analysis of OsMDR4 promoter activity in rice anthers and coleoptiles.

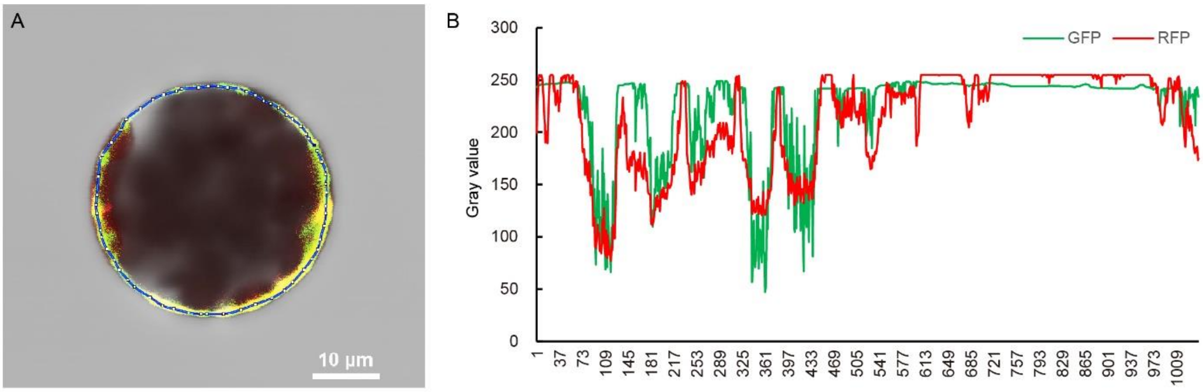

Figure S5. Line-scan fluorescence intensity analysis of OsMDR4-EGFP co-localization with the plasma membrane marker FM4-64.
